# Genomic Insights Into a Hospital‐Acquired High‐Risk Vancomycin‐Resistant *Enterococcus faecium* Outbreak in Guangdong, China

**DOI:** 10.1002/mbo3.70288

**Published:** 2026-04-13

**Authors:** Yang Guo, Chuyue Zhuo, Tianxin Zhang, Kuihai Wu, Zhengqi Shi, Yan Zhu, Jiayuan Huang, Chao Zhuo, Nan Qi

**Affiliations:** ^1^ State Key Laboratory of Respiratory Disease The First Affiliated Hospital of Guangzhou Medical University Guangzhou Guangdong China; ^2^ Department of Clinical Laboratory, Shenshan Medical Center, Sun Yat‐sen Memorial Hospital Sun Yat‐sen University Shanwei Guangdong China; ^3^ Department of Basic Research Guangzhou National Laboratory Guangzhou Guangdong China; ^4^ The First People's Hospital of Foshan Foshan Guangdong China; ^5^ Systems Biology Center, Tianjin Institute of Industrial Biotechnology, Chinese Academy of Sciences Tianjin China; ^6^ School of Medicine Shenzhen Campus of Sun Yat‐Sen University Shenzhen Guangdong China

**Keywords:** genomic analysis, molecular screening markers, multidrug resistance, public health surveillance, VR‐Efm

## Abstract

A comprehensive molecular epidemiological study was conducted on a vancomycin‐resistant *Enterococcus faecium* (VR‐Efm) outbreak in Guangdong, China, with the aim of analyzing transmission routes and antimicrobial resistance patterns, developing specific diagnostic tools for early detection, and elucidating genetic relationships with previously reported strains. Strain identification was performed using matrix‐assisted laser desorption/ionization mass spectrometry, whereas whole‐genome sequencing was performed for bacterial genomic characterization. Comprehensive bioinformatic analyses, including multilocus sequence typing, phylogenetic tree construction, principal component analysis, minimum spanning tree, and comparative genomic analyses, were performed. Our genomic investigation identified as predominant the sequence type 80 strain of VR‐Efm, which exhibited a high level of resistance to a range of antibiotics, particularly vancomycin, amoxicillin, and teicoplanin. Through genome sequencing, we established genetic proximity between the outbreak strain and those previously identified in India and Japan. Furthermore, functional genomic analyses have elucidated genetic variations within critical genes, such as *rsmH*, which may be associated with the acquisition of antibiotic resistance. The identification of unique molecular markers within the outbreak strain facilitated the development of specific PCR assays, thereby significantly enhancing our capacity for early and precise detection of VR‐Efm. Our in‐depth genomic analysis of the VR‐Efm outbreak in Guangdong, China, identified a predominant ST80 strain that exhibited multidrug resistance, especially to vancomycin. This finding underscores the need for enhanced global public health surveillance to address this emerging threat.

## Graphical abstract

We characterized a dominant ST80 clone responsible for a vancomycin‐resistant Enterococcus faecium outbreak in Chinese hospitals, revealing its close genetic ties to global lineages. A novel PCR method was developed for targeted surveillance of this high‐risk strain.

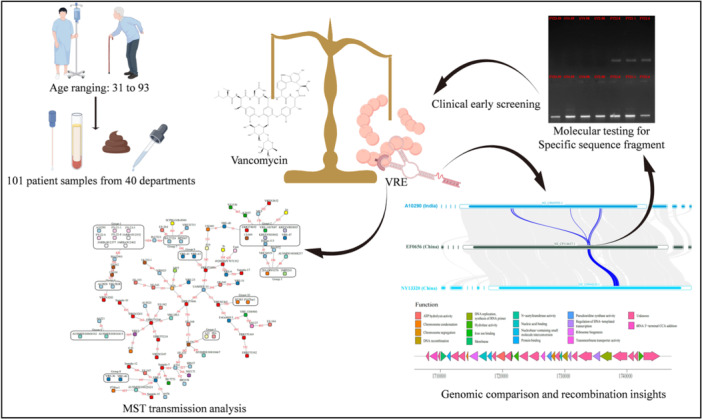

## 1 Introduction

Antimicrobial resistance is one of the most serious threats to human health in the 21st century. *Enterococcus faecium* is a prevalent nosocomial pathogen that poses formidable challenges in healthcare settings owing to its propensity to cause serious hospital‐associated infections (Higgs et al. 2022). Notably, these infections are accompanied by elevated levels of acquired antibiotic resistance, particularly against vancomycin (Tang et al. 2023; Fujiya et al. 2021). Mechanistically, vancomycin resistance commonly involves a reduction in the permeability of the bacterial cell wall to vancomycin, along with alterations in the cell wall synthesis pathway. These alterations prevent the effective binding of the antibiotic to its target, thereby reducing intracellular drug accumulation and conferring resistance (Pinholt et al. 2019). Managing hospital‐acquired vancomycin‐resistant *E. faecium* (VR‐Efm) infections presents a considerable challenge, largely attributable to elusive transmission routes (Fujiya et al. 2021; Pratama et al. 2022; Bender et al. 2022). Despite the implementation of stringent hospital infection control measures that have successfully reduced the dissemination of nosocomial pathogens, VR‐Efm has demonstrated a marked capacity for persistence within healthcare environments, thus imposing a substantial global burden (Kjær Hansen et al. 2021a; Benabbou et al. 2023; Kamus et al. 2022; Melese et al. 2020a).

Over the past decade, China has exhibited a lower prevalence of VR‐Efm infections than Europe and America. However, global outbreaks have garnered attention in the healthcare landscape, characterized by their persistence in hospital settings and rapid spread among vulnerable populations. These incidents lead to increased morbidity and mortality, a strained healthcare infrastructure, and effective antimicrobial therapies against multidrug‐resistant superbugs (Abdelbary et al. 2019; Vasconcelos et al. 2024; AL Rubaye et al. 2024). Containment of such outbreaks requires vigilant surveillance, stringent infection control measures, and concerted international efforts to mitigate antimicrobial resistance (Yan et al. 2023; Zhuo et al. 2024; Liu et al. 2022). According to the China Antimicrobial Resistance Surveillance System, the nationwide incidence of VR‐Efm was 1.2% in 2021 and increased to 1.7% in 2022 (http://www.carss.cn/). The the Indian ST80 reference isolate A10290 (GenBank ID: GCA_012933345.2), first identified in 2019 as part of a multidrug‐resistant ST80 lineage, carries a unique vanA‐positive plasmid and a divergent wzy gene cluster flanked by ISEf1/IS16 (Miller et al. 2003). Notably, Guangdong Province has witnessed a severe epidemic, with a substantial surge in prevalence from 1.5% in 2021 to 8.0% in 2022, which is significantly higher than the rates observed in all other regions of China (http://www.carss.cn/).

Increased antibiotic resistance across an integrated hospital network was evident. Between 2021 and 2023, clinical assessments at the First Affiliated Hospital of Guangzhou Medical University revealed an increasing prevalence of VR‐Efm cultures. The laboratory data for the VR‐Efm isolates showed increasing antibiotic resistance, including vancomycin resistance, during this timeframe. Concurrently, an outbreak was identified marked by a surge in VRE infection rates within hospitals across Guangzhou and Foshan over the last 2 years. Subsequent examination of medical records revealed an increased incidence of VR‐Efm clinical cultures in other health facilities during the same period, prompting concerns about inter‐facility transmission (Zhuo et al. 2024). Consequently, an investigation employing WGS was initiated to elucidate transmission dynamics and inform preventive measures.

In this study, we describe a novel VR‐Efm outbreak in Guangzhou and Foshan, characterized by its extensive transmission range, diverse infection spectrum, and multiplicity of antimicrobial resistance, which occurred from 2021 to 2023. To comprehensively characterize the outbreak VR‐Efm strains, we employed matrix‐assisted laser desorption/ionization (MALDI) mass spectrometry, antimicrobial susceptibility testing, and whole‐genome sequencing (WGS) of a cohort of 101 patient samples collected from 40 distinct departments in hospitals in Guangzhou and Foshan. Using genomic analysis, we elucidated the factors contributing to the outbreak, identified transmission routes, and explored measures to curb the spread of VR‐Efm. Our findings highlight the need for global action on public health vigilance and intervention strategies to inhibit the emergence and dissemination of VR‐Efm.

## 2 Materials and Methods

### 2.1 Study Design and Sample Characterization

#### 2.1.1 Study Design and Data Collection

This retrospective study was approved by the Research Project Review Ethics Committee of the First Affiliated Hospital of Guangzhou Medical University (Approval No: ES‐2023‐057‐01). Two hospitals in Guangzhou and Foshan were invited to participate in the study from March 2021 to November 2023, which resulted in the collection of 101 VR‐Efm isolates for WGS. Isolates were collected from either clinical or screening samples from inpatients at the First Affiliated Hospital of Guangzhou Medical University and The First People's Hospital of Foshan who stayed for more than 24 h. Written informed consent was obtained from all participants or their legal guardians prior to sample collection.

#### 2.1.2 VR‐Efm Identification

Clinical VR‐Efm isolates were identified using MALDI‐time of flight mass spectrometry with a Bruker BioTyper (Microflex LT; Bruker Daltonics GmbH, Bremen, Germany) (Ross et al. 2012; Saito et al. 2022).

#### 2.1.3 Antimicrobial Susceptibility Testing

Minimal inhibitory concentrations (MICs) were determined using the agar dilution or broth microdilution methods with cation‐adjusted Mueller‐Hinton broth. Interpretative breakpoint criteria were in accordance with those recommended by the Clinical and Laboratory Standards Institute guidelines and the European Committee on Antimicrobial Susceptibility Testing (Clinical and Laboratory Standards Institute 2024; Fujikura et al. 2019; Egan et al. 2020; Eisenberger et al. 2020; Rao et al. 2021). Nine antimicrobial agents were tested: vancomycin, ampicillin, gentamicin, streptomycin, linezolid, teicoplanin, rifampin, doxycycline, and daptomycin. Bacterial suspensions (> 10^4^ CFU for each bacterium) were obtained using a multipoint inoculator. *E. faecium* ATCC 29212 was selected as the quality control reference strain. The detailed MIC interpretive criteria for each antimicrobial agent are provided in Supplementary Table S3.

### 2.2 Genomic Data Generation and Phylogenetic Analysis

#### 2.2.1 DNA Sequencing Pipeline

##### 2.2.1.1 DNA Extraction and Library Construction

**Figure 1:**
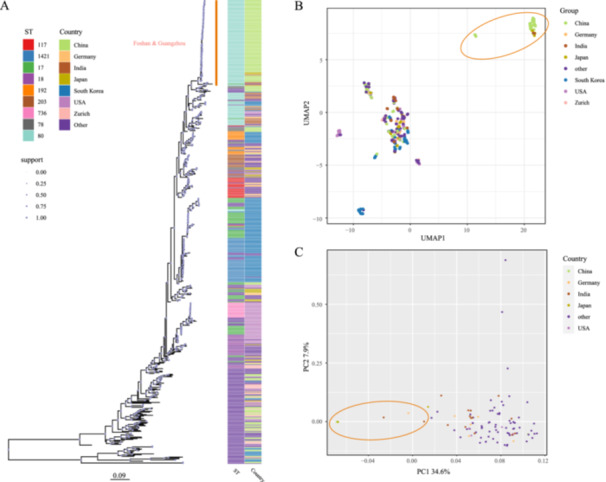
Genomic analysis of bacterial strains reveals close phylogenetic relationships among strains of different origins. (A) Phylogenetic tree was constructed using the Maximum Parsimony method, illustrating the evolutionary relationships among the strains. (B) UMAP clustering analysis depicting the genetic similarity and distinct clusters formed by the strains. (C) PCA plot highlighting the genomic variation among the strains along the first two principal components. Each strain is represented by a unique symbol/color, and the proximity of the symbols/clusters in the UMAP and PCA plots indicates the degree of relatedness. PCA, principal component analysis; UMAP, uniform manifold approximation and projection for dimension reduction.

Bacterial genomic DNA was extracted from clinical samples using The E.Z.N.A. Stool DNA Kit (D4015‐02, Omega, USA) according to the manufacturer's instructions (Figure 1). Whole‐genome sequencing was performed using the Illumina NovaSeq. 6000 platform (paired‐end, 150 bp). The DNA library was constructed using a TruSeq Nano DNA LT Library Preparation Kit (FC‐121‐4001) according to the manufacturer's protocol (LC‐Bio Technologies, Hangzhou, Hang Zhou, Zhejiang Province, China).

##### 2.2.1.2 Raw Data Processing and Assembly

Raw sequencing reads were processed using fastp v0.23.4 for adapter removal, quality analysis, and low‐quality read trimming with default parameters. Reads were aligned to the human reference genome, GRCh38, using BWA v0.7.17‐r1188 to detect host reads, which were marked and removed using SAMtool v1.17. Single nucleotide polymorphisms (SNPs) and indels were called using Snippy v4.6.0 with default parameters to remove low‐quality SNPs and create vcf format files. Quality‐trimmed reads were reserved and input into SPAdes v3.15.5 for genome assembly (van Hal et al. 2022).

We note that our inferences on large‐scale genomic rearrangements are based on short‐read sequencing. While this approach reliably identifies SNPs and small indels, the precise boundaries and nature of putative recombination events, suggested by patterns such as coverage drops or synteny breaks, remain to be confirmed by long‐read technologies.

#### 2.2.2 Strain Typing and Evolutionary Inference

##### 2.2.2.1 Multilocus Sequence Typing (MLST) and Core Genome MLST (CgMLST)

In addition to the 101 isolates collected in this study, we utilized an additional 476 sequences from the National Center for Biotechnology Information (NCBI) database. All 577 sequences underwent in silico MLST using the MLST tool (v2.23.0) to determine their sequence type (ST). The tool automatically applied the standard multilocus sequence typing scheme for *Enterococcus faecium* from the PubMedST database (https://pubmlst.org/organisms/enterococcus-faecium). For cgMLST, ChewBBACA v3.3.3 was used to create an *E. faecium* scheme with the default pipeline and assess protein types and allelic locus distances in different samples (Chuang et al. 2018; Pidot et al. 2018).

The analysis followed the software's recommended workflow. Briefly, a core genome schema was created from reference genomes using the CreateSchema module. Allele calling for all study isolates was then performed using the AlleleCall module with default parameters, including a BLAST score ratio threshold of 0.6 for paralog detection (‐‐ptf 0.6). The schema and allele calls were subsequently refined using the SchemaEvaluator and AlleleCallEvaluator modules. Finally, a core genome MLST (cgMLST) matrix was extracted using the ExtractCgMLST module, considering loci present in ≥ 99% of isolates as core.

##### 2.2.2.2 Phylogenetic Analysis

kSNP4 (https://github.com/kissake/kSNP4) was used to identify the SNPs in the *E. faecium* population and use them to create parsimony phylogenetic trees with the parameters ‐k 21 ‐vcf ‐core ‐min_frac 0.75. This pipeline was used for phylogenetic analysis and encompassed 577 sequences from organisms isolated in China (52), Japan (24), India (21), Australia (16), and other countries (464). Iqtree v2.2.6 was also used to create phylogenetic trees with the parameters ‐m MFP. The trees constructed using kSNP4 were visualized and marked using the R package ggtree v3.6.2 (Freitas et al. 2016). Given the focus on a short‐term, clonal hospital outbreak (ST80), we did not apply dedicated recombination filtering (e.g., using Gubbins or ClonalFrameML) prior to phylogenetic inference. The impact of recent recombination on the major topology of the core‐genome phylogeny is expected to be limited under these conditions, and the primary conclusions regarding clonal expansion are robustly supported by congruent results from minimum spanning tree (MST) and cgMLST analyses.

##### 2.2.2.3 Uniform Manifold Approximation and Projection for Dimension Reduction (UMAP) and Principal Components Analysis (PCA)

The cgMLST profiles of the 577 samples were visualized in two dimensions using the UMAP function of uwot package of R with default parameters (Tacconelli and Cataldo 2008; Melese et al. 2020b). To unveil the potential transmission relationships of the samples, PCA analysis using VCF2PCACluster v1.40 was performed with default parameters on all ST80‐type VR‐Efm present in the database. SNPs identified by kSNP4 were used for PCA (Werner et al. 2020).

##### 2.2.2.4 MST and Transmission Cluster Analyses

The core SNPs of ST80‐type‐specific VR‐Efm sequences identified using kSNP4 were used to construct the MST using GraphSNP v1.0 with the default parameters. With detailed country and data information, GraphSNP was used to perform transmission cluster analysis.

##### 2.2.2.5 Time Trees and Transmission Event Analysis

To obtain a robust temporal signal for time‐calibrated phylogenetic inference, a filtering strategy was applied to the initial 101 isolates. First, to reduce clonal redundancy resulting from recent nosocomial transmission, isolates were clustered based on core‐genome SNP differences (≤ 10 SNPs), and only the earliest sampled representative from each cluster was retained. Subsequently, an iterative screening was performed based on the relationship between sampling dates and root‐to‐tip genetic distances, removing outliers that significantly deviated from the linear regression trend. This process yielded 31 isolates that exhibited a significant temporal signal (*R*² = 0.83, *p* < 0.0001, see Figure S2) and were used for subsequent Bayesian time‐tree estimation.

Phylogenetic trees were generated using BactDating v1.1.2 under a mixedcarc model. Markov Chain Monte Carlo (MCMC) analysis was run for 10^6 iterations. Model convergence was assessed by monitoring the effective sample size (ESS) for key parameters. The ESS for the substitution rate (*μ*) was 801, and the ESS values for other parameters (e.g., *α*, *σ*) all substantially exceeded the conventional threshold of 200, indicating excellent chain convergence and reliable inference (Figure S3). Time trees created with BactDating were input into TransPhylo version 1.3.2 to generate transmission trees and evaluate the role of staff versus resident introduction and transmission events.

### 2.3 Population and Functional Genomic Profiling

#### 2.3.1 Genomic Variation and Population Structure

##### 2.3.1.1 Variant Calling and Coverage Rate Analysis

The ST80 isolate A10290 was selected as the primary reference for read alignment due to its high‐quality, complete genome assembly and its close genetic and phenotypic relevance (ST80, vanA‐positive) to our outbreak strains. To mitigate potential bias from using a single reference, we also performed reference‐free phylogenetic and clustering analyses using kSNP4 and cgMLST, respectively, which yielded congruent results (see Results). BWA was used to align filtered reads to the Indian reference genome, A10290, and the resulting BAM files were used to analyze the SNPs using bedtools to perform population genomic analysis. SNP calls for core SNP visualization and function analysis were performed using Snippy v4.6.0. Bedtools v2.30.0 was used to calculate the coverage rate of the samples (MacDougall et al. 2020).

##### 2.3.1.2 Population Genomic Analysis

Plink v1.90b6.21 was used to evaluate linkage disequilibrium to select representative SNPs with the parameters ‐‐indep‐pairwise 50 10 0.2 (Smout et al. 2023). Admixture v1.3.0 was used to estimate individual ancestries with the parameters ‐j2 ‐C 0.01 –cv and the outcome with the lowest cv value was selected.

#### 2.3.2 Comparative Genomics and Functional Annotation

##### 2.3.2.1 Genomic Comparisons

Mauve (v2.3.1) was used to align the complete chromosomes of *E. faecium* A10290, *E. faecium* A11051, *E. faecium* JARB‐OU2352, *E. faecium* VB12993, and *E. faecium* VB13828 with default parameters to reveal the genetic relationship between samples from different countries (Kjær Hansen et al. 2021a).

##### 2.3.2.2 Synteny Analysis

Tbtools was used to perform synteny analysis using the functions Quick Run MCScanX Wrapper and Multiple Synteny Plot to display the relationship among the whole genomes of *E. faecium* A10290, *E. faecium* EF0656, and *E. faecium* NY13320 isolated from India and China.

##### 2.3.2.3 Kyoto Encyclopedia of Genes and Genomes (KEGG) Pathway Analysis

Kofamscan was used to analyze the function of proteins predicted by Prokka with the parameters ‐f mapper ‐E 1e‐3 and provide ko ids to proteins. KEGG dot plots were generated using the enrichKEGG function provided by the clusterProfiler v4.10.0 package, and Venn diagrams were created using Tbtools (Simshauser et al. 2022).

##### 2.3.2.4 Pan‐Genome, Resistance, and Virulence Analyses

We estimated the pan‐genome using contigs assembled with SPAdes. Contigs and other sequences from Japan and India were annotated using Prokka before pan‐genome discovery using OrthoFinder v2.5.5 with the parameters ‐S diamond ‐og, and were used for pan‐genome analysis. Orthogroups obtained using OrthoFinder were sampled for subsequent pan‐genome analysis in R. The nlsLM function provided by minpack.lm v1.2‐4 was used to calculate the fit curve to evaluate the pan‐genome type of the India‐related ST80 VR‐Efm (Cassone et al. 2023a). Contigs and other sequences were analyzed for antimicrobial virulence and resistance genes using the Virulence Factor Database and card databases provided by Abricate v1.0.1. The outcomes of resistance and virulence analyses were integrated using the function Abricate ‐summary to identify the relevant genes in the database.

### 2.4 Experimental Validation and Data Visualization

#### 2.4.1 Molecular Biology Testing

Total genomic DNA was extracted from 101 isolates using the Qiagen DNeasy Blood & Tissue Kit (Qiagen, Hilden, Germany) following the manufacturer's protocol. DNA concentration and purity were assessed using a NanoDrop 2000 Spectrophotometer (Thermo Fisher Scientific, Waltham, MA, USA), and the samples were normalized to a final concentration of 50 ng/μL. Primers for the target genes were designed using BLAST and synthesized by Sangon Biotech (Shanghai). We designed a pair of specific primers targeting the structurally divergent region at approximately 2.1 million base pairs (bp) (described above) in the genome of the outbreak‐associated *E. faecium* ST80 strain. The universal bacterial primer, *16S rRNA*, was used as a control. We then selected four non‐ST80 VRE‐fm and three ST80 VRE‐fm samples from the 101 cases and performed PCR amplification. The VR‐Efm primer pair sequences were as follows: forward primer, 5′‐AATCAGACGATCAAGATTGGAACAAC‐3′; reverse primer, 5′‐AAGGATTCATCTCATCCGTTGCG‐3′. The universal bacterial primer, *16S rRNA*, were as follows: 27F primer, 5′‐AGAGTTTGATCCTGGCTCA‐3′; 1492R primer, 5′‐GGTTACCTTGTTACGACTT‐3′.

#### 2.4.2 Data Visualization and Statistical Analysis

Figures were generated in R (v4.0.2, https://www.R-project.org/) using Tidyverse, ggplot2, ggtree, ggpubr, ggextra, ggsci, and a Complex Heatmap. The coefficient of the fit curve was calculated using minpack lm based on an NLS regression model.

## 3 Results

### 3.1 Collection of Clinical VR‐Efm Isolates

A total of 101 VR‐Efm isolates (Tables 1 and S1) were collected from 101 patients admitted to the First Affiliated Hospital of Guangzhou Medical University between March 2021 and November 2023. Analysis of the demographic information for these patients (54 males and 47 females) revealed an age range of 25 to 93 years (68.27 ± 16.08 years). The isolates were predominantly obtained from urine (66, 65.3%) and sputum (11, 10.9%) samples, with others isolated from ascitic fluid, wound swabs, blood, and other sources (see Table 1 for a complete breakdown). In the patient cohort, 38 individuals were from 12 departments of Guangzhou Hospital and 63 patients were from 28 departments of Foshan Hospital.

**Table 1:** Isolate statistics.

VRE‐fm^a^ isolate collection location	
Guangzhou	38 (37.62％)
Foshan	63 (62.38％)
Patients age ranges	
Median(IQR)	68 (56–77)
≤ 40	9 (8.91％)
41–50	5 (4.95％)
51–60	13 (12.87％)
61–70	20 (19.80％)
71–80	33 (32.67％)
≥ 81	21 (20.79％)
VRE‐fm isolate specimen source	
Bile	3 (2.97％)
Sputum	11 (10.89％)
Stool	1 (0.99％)
Urine	66 (65.35％)
Ascitic Fluid	7 (6.93％)
Catheter Site	2 (1.98％)
Cerebrospinal Fluid	1 (0.99％)
Secretion	1 (0.99％)
Pleural Effusion	1 (0.99％)
Fluid	2 (1.98％)
Blood	2 (1.98％)
Wound Swab	4 (3.96％)
VRE‐fm isolate sequence types	
ST80	97 (96.04％)
ST78	2 (1.98％)
ST988	1 (0.99％)
ST547	1 (0.99％)
Total	101

^a^ VR‐Efm, vancomycin‐resistant *Enterococcus faecium*.

^b^ IQR, interquartile range.

### 3.2 Genome Sequencing and Assembly

Genome sequencing of the 101 VR‐Efm isolates yielded a robust dataset, with each sample yielding 2.5–10.2 million reads. After filtering, 2.08–2.78 million quality‐trimmed reads were obtained for each sample, with an average read length of 129–150 bp and representing over 100‐fold coverage of the genomes. De novo assembly yielded 149–198 contigs (≥ 1000 bp) for each isolate, collectively spanning a total length of 2,792,348 to 3,128,009 bp. The GC content fell within 37.34%–37.8%, with N50 ranging from 31,195 to 40,661 bp. Overall, 2,717‐3,017 protein‐encoding sequences were annotated from the reference genome (A10290) using Prokka software v.1.14.5.

### 3.3 Antimicrobial Resistance

MIC testing revealed that all 101 isolates were resistant to vancomycin (VAN), amoxicillin (AMX), and teicoplanin (TEI). As shown in Table 2, resistance rates to daptomycin (DAP), rifampicin (RIF), and gentamicin (GEN) were 43.56%, 99.01%, and 93.07%, respectively. All the isolates were susceptible to streptomycin (STR). All strains were susceptible to linezolid (LZD), with only one exception (GV 4‐95).

**Table 2:** Antimicrobial agent susceptibilities of 101 VR‐Efm Isolates.

	No. (％) of isolates with drug susceptibility			MIC(μg/mL)^b^		
Antimicrobial agent	S^a^	I^a^	R^a^	S	I	R
Vancomycin			101 (100)	≤ 4	8–16	≥ 32
Ampicillin			101 (100)	≤ 8		≥ 16
Gentamicin	7 (6.93)		94 (93.07)	≤ 500		＞500
Streptomycin	101 (100)			≤ 1000		＞1000
Linezolid	97 (96.04)	2 (1.98)	1 (0.99)	≤ 2	4	≥ 8
Teicoplanin			101 (100)	≤ 8	16	≥ 32
Rifampin	1 (0.99)		100 (99.01)	≤ 1	2	≥ 4
Doxycycline	22 (21.78)	66 (65.35)	13 (12.87)	≤ 4	8	≥ 16
Daptomycin	57 (56.44)		44 (43.56)	≤ 4		≥ 8

*Note:* The minimum inhibitory concentration (MIC) for drug susceptibility testing is based on reference literature (Miller et al. 2003; Ross et al. 2012; Saito et al. 2022).

^a^ S, susceptible; I, intermediate; R, resistant.

### 3.4 Genetic Relationships Between VR‐Efm Isolates From the Outbreak and Other Countries

To reveal the transmission and evolutionary relationships between the VR‐Efm isolates from the current outbreak and those from other countries, we conducted a phylogenetic analysis. A phylogenetic tree constructed using the maximum parsimony method revealed that the genomes of VR‐Efm from recent outbreaks in Guangzhou and Foshan, China, were evolutionarily clustered with VR‐Efm from India and Japan within the same clade and were the most closely related (highlighted area in Figure 1A). Analysis of the genetic profiles of samples from various countries using cgMLST, followed by cluster analysis using UMAP, showed that most samples were located at the same position as the Japanese and Indian strains (circled areas in Figure 1B). The results revealed that of the 101 strains of VR‐Efm in this study, over 96% were ST80 (*n* = 97), two were ST78, one was ST988, and one was ST547. We then performed PCA of all ST80 samples, comprising different countries, isolation times, and STs defined by the new multilocus sequence typing scheme (Bezdicek et al. 2023). The results indicated that the Japanese samples were positioned in the same area as the Chinese outbreak strains, suggesting smaller differences, whereas the A10290 (India), BA7523 (India), and P79Iso1 (Germany) strains were positioned a certain distance from the outbreak strains (circled area in Figure 1C). This genetic proximity suggests that the outbreak strains and A10290 share a recent common ancestor within the globally circulating ST80 lineage. A summary of sequence typing and assembly quality control of the 101 VR‐Efm strains from the current outbreak is shown in Table S2.

### 3.5 MST Transmission Analysis

**Figure 2:**
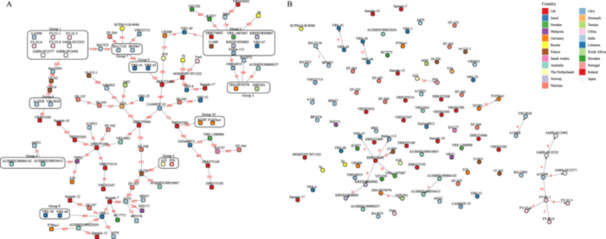
MST transmission analysis. (A) The Indian strain A10290, the Guangzhou outbreak strains (FV‐21‐1, FV‐22‐6, FV‐22‐8, and FV‐23‐5), and Japanese strains (JARB‐OU2352, JARB‐OU2377, and JARB‐OU2402) are labeled as Group 1, which exhibits a potential transmission link. (B) Provides an expanded view of the MST, highlighting the Indian strain, A10290, in the lower right corner, which is connected to several isolates. This central position of A10290 may signify its role as a transmission hub and could reveal a pivotal transmission event. MST, minimum spanning tree.

MST transmission cluster analysis was performed based on SNP differentiation of ST80‐type VR‐Efm strains from the current outbreak and all available ST80 strains in the NCBI database. To reduce redundancy in the correlation analysis of the strains regarding their coverage with A10290, we selected representative strains that exhibited recombination and collected them for further analysis. These results are consistent with those obtained from the analysis of multiple strains. Figure 2A presents the MST, with the upper left corner enclosed in a box labeled Group 1, which includes the Indian reference isolate A10290, Guangzhou outbreak strains (FV‐21‐1, FV‐22‐6, FV‐22‐8, and FV‐23‐5), and Japanese isolates (JARB‐OU2352, JARB‐OU2377, and JARB‐OU2402). The results indicated that the strains from the recent Guangzhou outbreak are most closely related to strains from India and Japan, suggesting a potential transmission link because they share notable similarities in their genomic composition. Figure 2B provides an expanded view of the MST, highlighting the Indian strain, A10290, in the lower‐right corner, which is connected to several isolates. The central position of A10290 within the genetic network underscores its close genetic relatedness to many isolates from the outbreak and to strains from Japan. MST serves as a visual representation of the genetic relationships and potential transmission pathways among ST80‐type VR‐Efm strains, with the country of origin of each strain annotated accordingly.

### 3.6 Time‐Dated Phylogenetic Trees

**Figure 3:**
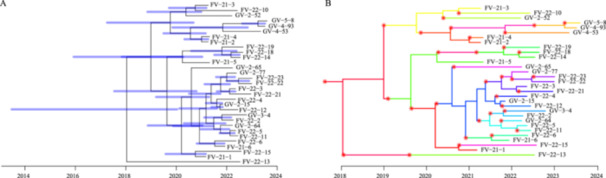
Time‐dated phylogenetic analysis. (A) Time‐dated phylogenetic tree with internal nodes annotated. Bars indicate the 95% highest posterior density intervals. (B) A root‐to‐tip analysis to estimate the time since the most recent common ancestor. The nodes indicate the time point of sampling. The asterisks represent transmission events and correspond to panel A.

To further pinpoint the evolutionary timeline of the VR‐Efm outbreak isolates, 31 sequences (selected based on the temporal signal filtering criteria described in Section 2.2.2) were analyzed. Evolutionary timelines of VR‐Efm outbreak isolates and putative transmission routes that contributed to the evolution of the outbreak strain were evaluated. Figure 3A shows the estimated time of the most recent common ancestor, indicating that it existed between 2018 and 2022. This timeframe is similar to that of the discovery of strains in India and coincides with the time range (2021–2023) of our isolates. Figure 3B shows the transmission of outbreak strains with a 95% confidence interval, illustrating the potential spread of the sampled strains over time. The lineage marked as FV‐21‐1 to FV‐22‐23 and GV‐2‐52 to GV‐5‐8 are indicated in chronological order of their discovery from 2014 to 2024, which represents the period of the emergence of the common ancestor of the strains. Phylogenetic analysis suggests a close temporal association with Indian strains, indicating a possible epidemiological link.

### 3.7 Structural Variation Analysis

**Figure 4:**
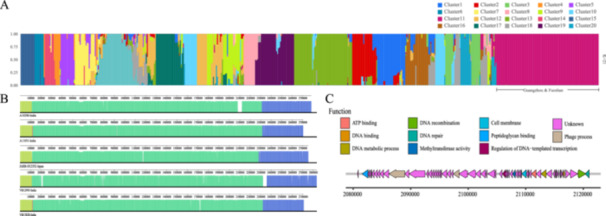
Genomic structure analysis of outbreak strains revealing structural homology and functional insights. (A) The analysis indicates a clear genetic homogeneity between the outbreak strain in question and strains originating from Japan and India, suggesting a shared ancestry or recent gene flow between these populations. (B) Mauve analysis. A notable structural congruence is observed among the Indian strain, A10290, the Japanese strain, JARB‐OU2352, and three other Indian strains (A11051, VB12993, and VB13828). However, a significant region of difference is highlighted around the 2,100,000 base pair (bp) marker, indicating potential genetic recombination or the presence of a unique genetic element in this region. (C) The functional annotation provides insights into the potential biological implications of the genetic differences observed in the Mauve analysis.

To further identify potential variations within the microbial community structure that may affect outbreaks and transmission, we performed structural variation analysis. Figure 4A shows the results of the structural analysis, where the far right of the image displays the strains from recent outbreaks in Guangzhou and Foshan. The similarity in coloration between these strains, and those from Japan and India, suggests a resemblance in the ancestral structure of the strains, indicating that the outbreak strain from the current epidemic shares population structure and evolutionary connection with strains originating from Japan and India.

Figure 4B shows a mauve analysis of multiple sequence similarities, revealing that the Indian strain A10290 exhibited significant structural homology with other sequences, specifically the Japanese strains (JARB‐OU2377, JARB‐OU2352, and JARB‐OU2402) and three Indian strains (A11051, VB12993, and VB13828). Notably, patterns suggestive of a major genomic rearrangement, including a sharp drop in sequencing coverage and a break in synteny, were identified near the 2,100,000 bp locus. This region was characterized by a dramatic drop in sequencing coverage when aligned to the reference genome A10290, indicating lineage‐specific structural variation or high sequence divergence. This distinct genomic architecture served as a stable molecular signature distinguishing the outbreak clone. The genomic variation observed at the 2,100,000 bp locus in the Indian strain A10290 is likely a recent event, as it was not detected in the other sequences. This indicates that the A10290 strain did not represent the earliest ancestral form within this lineage. Conversely, it may represent a more recently diverged branch in the evolutionary tree, suggesting later emergence in the phylogenetic history of the strain. Figure 4C shows the functional annotation of the genomic region near the 2,100,000 bp region in strain A10290, highlighting the genetic functions within this divergent area. The identified functions include ATP binding, DNA binding, DNA metabolic processes, DNA recombination, DNA repair, methyltransferase activity, cell membrane, peptidoglycan binding, and regulation of DNA‐templated transcription. These results indicate that although the Indian strain A10290 has a clear phylogenetic relationship with the previous Japanese outbreak strain, as well as with the strains from the current outbreaks in Guangzhou and Foshan. These genomic distinctions indicate that while closely related, A10290 is not the direct progenitor of the outbreak strains sequenced in this study, highlighting the genomic diversity within the ST80 lineage. However, certain genomic differences exist between the two species.

### 3.8 SNP and Coverage Analysis

**Figure 5:**
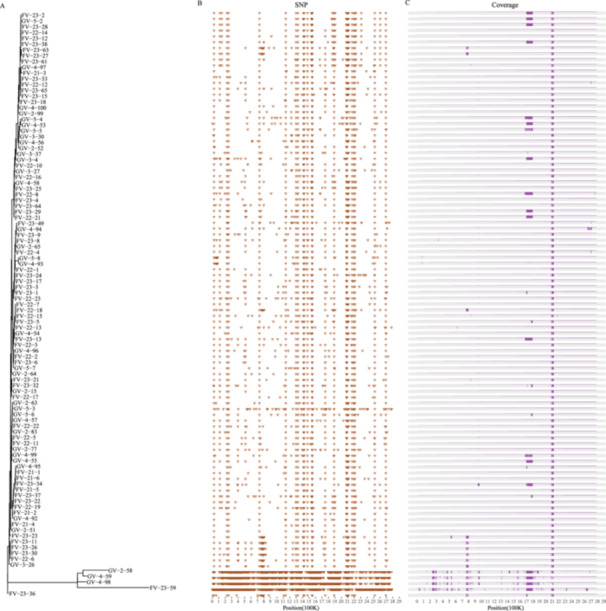
Genomic SNP analysis and coverage insights into the evolutionary proximity of the outbreak VR‐Efm and Indian strain A10290. (A) Phylogenetic tree analysis of 101 strains of VR‐Efm. (B) Genomic analysis comparing the SNPs. The SNP analysis reveals a close genetic relationship between the outbreak strains and the Indian strain A10290, with some strains exhibiting fewer than 100 SNP differences, suggesting a recent evolutionary divergence. Each dot or color intensity represents an SNP. (C) Sequence coverage analysis comparing the outbreak VR‐Efm strains with the Indian reference strain A10290. The *y*‐axis represents the coverage depth, while the *x*‐axis is marked in increments of 100,000 bp (position 100 K), providing a scale for genomic locations. Pronounced drops in coverage depth (e.g., near the ~2.1 Mb region, as indicated) typically suggest local sequence absence or high divergence in the outbreak strains relative to the reference, potentially resulting from recombination or deletion events. Conversely, peaks may indicate duplications or insertions. The coverage variations highlight genomic segments that may have undergone structural rearrangements.

To elucidate the genetic relationships and genetic differences among strains, we performed SNP and coverage analyses. Figure 5A shows the phylogenetic tree analysis of the 101 strains of VR‐Efm. The four strains at the bottom of the image (GV‐2‐58, GV‐4‐59, GV‐4‐98, and FV‐23‐59) were segregated into distinct clusters, indicating that they were different from the majority of strains involved in this outbreak. These isolates were identified as ST78, ST988, and ST547, respectively, which is consistent with the typing results presented in Figures 1A and 5A. In conjunction with the ST typing results (Figure 1A), these four strains corresponded to ST78, ST988, and ST547, respectively, and they exhibited a significant number of SNPs that differentiated them from the remaining 97 ST80 strains (Figure 5B). Figure 5B displays the SNP analysis, highlighting the genetic differences between the outbreak strains and the Indian strain A10290. For the vast majority of the strains involved in this outbreak, the corresponding SNP sites were relatively fixed and the number was comparatively limited. Some strains had fewer than 100 SNPs compared with those in A10290, which suggests a relatively recent evolutionary split from this Indian strain. Figure 5C shows the coverage analysis, which is crucial for identifying regions of the genome that may have undergone recombination or other genetic alterations. The *y*‐axis represents the coverage depth, whereas the *x*‐axis is marked in increments of 100,000 bp (position 100 K), providing a scale for genomic locations. Variations in coverage depth may indicate areas of the genome with significant genetic changes, such as those resulting from recombination. Although the outbreak strains were closely related to the Indian strain A10290 at the SNP level, some genomic regions of the Chinese strains exhibited signs of recombination, as evidenced by coverage analysis (Figure 5). Genomic plasticity is suggested by patterns consistent with potential recombination (e.g., coverage variations), which may have contributed to the strain's adaptation during the outbreak.

### 3.9 SNP and Functional Genomic Analyses

**Figure 6:**
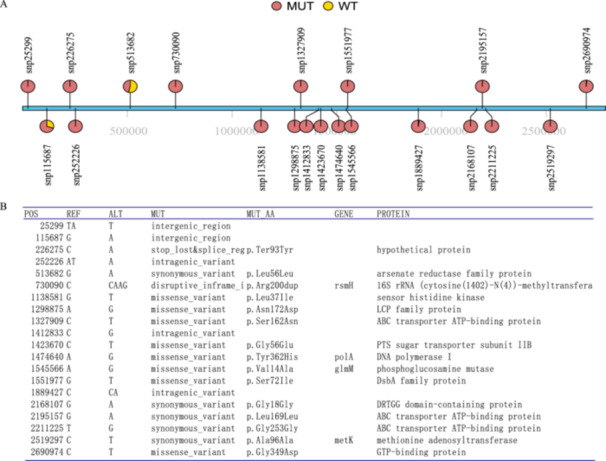
SNP and functional genomic analysis of outbreak strain versus Indian strain A10290. (A) The figure illustrates the specific SNP positions present in the outbreak strain of interest and their associated gene functions, in comparison with the Indian strain, A10290 (referred to as WT for wild type). The mut designation indicates the mutations found in the outbreak strain, while WT represents the reference genotype of the Indian strain, A10290. The blue line represents the annotated genome of the Indian strain, A10290, and circles along this line denote the annotated SNPs. (B) The figure lists the SNPs identified in the outbreak strain compared to the Indian strain A10290, including their positions, reference and alternate alleles, mutation types, amino acid changes, affected genes, and associated proteins. SNP, single‐nucleotide polymorphism.

To delineate the genetic underpinnings of the outbreak strain compared to the Indian strain, A10290 (our reference wild type), we conducted a comprehensive genomic analysis. Figure 6A shows a detailed examination of the SNPs across the genomes of the outbreak and reference strains. The color composition indicates the proportion of the sequence that matched the A10290 strain. Orange represents a higher proportion of the sequence that aligns with the A10290 strain, whereas red indicates a lower proportion of alignment. This visual representation aids in rapid assessment of the conservation and potential functional implications of these SNPs, offering insights into the genetic variations that may contribute to the distinct characteristics and pathogenicity of the outbreak strain. The figure displays SNPs unique to the outbreak strain (“MUT”) and their effects compared to the A10290 strain (“WT”). The blue line indicates the reference genome with the SNPs marked along it. These SNPs were plotted along the *x*‐axis, which was scaled to reflect their genomic positions, whereas the *y*‐axis shows SNPs based on their functional impacts on various genes and gene products. Notably, Figure 6B includes annotations that specify the type of genetic variation present at each SNP site, such as missense, synonymous, and intragenic variants, and the potential protein‐level changes that may result from these mutations. We also annotated the biological functions of the genes harboring these SNPs. Notably, a missense mutation was identified in the rsmH gene, which encodes a 16S rRNA (cytosine(1402)‐N(4))‐methyltransferase—an enzyme class implicated in resistance to ribosome‐targeting antibiotics such as tetracyclines (Zou et al. 2020). SNPs were also found in genes encoding a sensor histidine kinase (a component of two‐component signaling systems), an LCP family protein, and an ABC transporter ATP‐binding protein, all of which are involved in various aspects of bacterial stress response, cell wall metabolism, and adaptation. The phenotypic or fitness impacts of these specific SNPs were not assessed in this study and remain to be functionally characterized. This genomic comparison provides a foundation for understanding the molecular mechanisms underlying the pathogenic potential of the outbreak strains.

### 3.10 Genomic Comparison and Recombination Analysis

**Figure 7:**
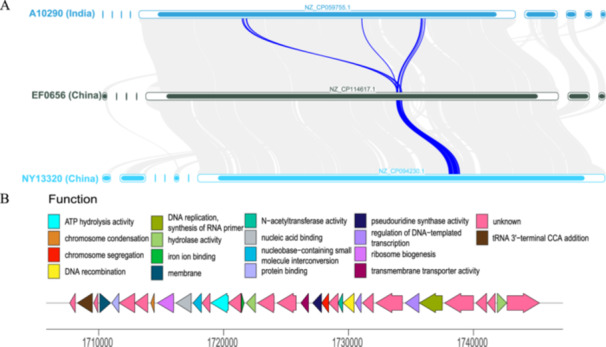
Genomic comparison and recombination analysis of outbreak strain, EF0656, with the Indian (A10290) and Chinese (NY13320) strains. (A) A comparative analysis of the outbreak strain, EF0656, from China, using third‐generation sequencing, against the Indian strain, A10290, and another Chinese strain, NY13320. The analysis focuses on a specific region (170,000–175,000 bp) where strain EF0656 exhibits significant divergence from A10290 but shares high homology with NY13320, suggesting potential recombination events within this region. (B) The schematic illustrates the genomic architecture and highlights the functional categories of genes located in this region, which include ATP hydrolysis activity, chromosome condensation, DNA recombination, and various other essential cellular processes.

To compensate for the gap in our third‐generation sequencing, we utilized a strain of VR‐Efm‐EF0656 previously obtained in Zhuhai, whose genomic sequence was very similar to that of the outbreak strains in our current study. Therefore, we conducted a synteny analysis using this strain, along with the Indian strain A10290 and the Chinese strain NY13320. Figure 7A shows a detailed genomic comparison between the Chinese outbreak strain EF0656, two reference strains, the Indian strain A10290, and the Chinese strain NY13320. Using third‐generation sequencing data, the analysis was focused on a critical genomic segment spanning positions 1,700,000 to 1,750,000 bp, where genetic distance was observed between EF0656 and A10290, and a notable alignment with NY13320 was detected. The high degree of similarity between the two Chinese strains in this region suggests a potential for genetic recombination. Our current strains are more closely related to the Chinese strain EF0656 than to the Indian strain A10290, confirming that the outbreak strains have evolved to carry genomic mutations with Chinese characteristics.

Moreover, we categorized the functional genes located within a specified region based on their biological functions with KEGG. Genes implicated in essential processes, such as ATP hydrolysis, chromosome condensation, DNA recombination, and other cellular activities, are depicted (Figure 7B). This functional genomic annotation aids in determining the biological implications of the observed recombination, which may be integral to the pathogenicity and adaptation of the VR‐Efm strain.

### 3.11 Genomic Comparison and Recombination Analysis

**Figure 8:**
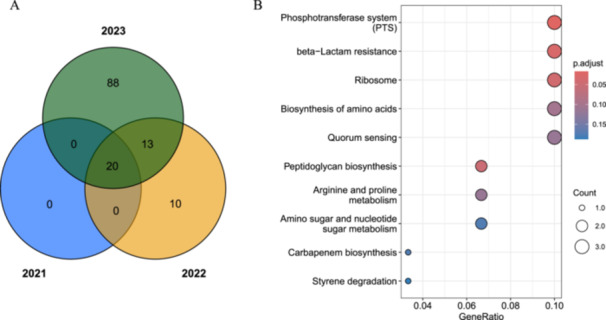
Annual SNP accumulation and functional enrichment of SNPs in Chinese isolates compared to Indian strain A10290. (A) Venn diagram, the annual count of SNPs that have emerged in the bacterial strain since its introduction to China, plotted over consecutive years. Each data point represents the number of SNPs identified in the respective year, reflecting the strain's genetic evolution over time. (B) The functional enrichment analysis of SNPs identified in the years 2022 and 2023 when compared to the Indian strain, A10290. The analysis reveals significant associations with genes involved in beta‐lactam resistance and quorum sensing, highlighting the potential adaptive changes in the bacterial strain that may confer resistance and influence communication mechanisms within the bacterial population. The figure is a dot plot representation, where the *x*‐axis represents the gene ratio, and the *y*‐axis denotes the different functional categories. The enrichment analysis provides insights into the functional implications of the observed SNPs and their potential role in the bacterial strain's pathogenicity and resistance profile. SNP, single‐nucleotide polymorphism.

We analyzed a dual perspective on the genomic evolution of the bacterial strain in question after its introduction into China. Figure 8A shows the incremental accumulation of SNPs (in at least two samples) over subsequent years, offering a temporal snapshot of the genetic trajectory of the strain. This annual tabulation of SNPs not only charts genetic drift but also underscores the dynamic nature of bacterial adaptation. SNP accumulation shows a continuously increasing trend over time. Conversely, Figure 8B shifts the focus to a functional enrichment analysis of the SNPs identified in 2022 and 2023, in juxtaposition with the Indian strain A10290. This comparative approach yielded notable insights, particularly regarding the significant enrichment of genes associated with beta‐lactam resistance and quorum sensing. The correlation with beta‐lactam resistance is of particular concern as it may signal the emergence of antibiotic resistance mechanisms. Additionally, quorum sensing suggests potential regulatory changes in bacterial communication that could affect strain virulence and coordination. A dot plot encapsulates these findings, with the *y*‐axis representing the spectrum of functional categories affected by the SNPs and the *x*‐axis illustrating the gene ratio, frequency of occurrence, and statistical rigor of the enrichment (p.adjust). This visual synthesis of data not only provides a comprehensive overview of the genetic changes but also serves as a springboard for further investigation into the mechanistic underpinnings of these adaptations. The identification of SNPs linked to beta‐lactam resistance warrants vigilant monitoring and underscores the need to develop novel therapeutic strategies.

This temporal increase in SNPs provides valuable insights into the genetic alterations that have transpired within the lineage, reflecting the dynamic nature of bacterial evolution. Moreover, it may indicate the molecular mechanisms underlying adaptive advantages, such as antimicrobial resistance. Furthermore, the observed association with quorum sensing opens avenues for exploring targeted interventions that can disrupt bacterial coordination mechanisms. Notably, the p‐adjusted value for quorum sensing was 0.12, which, although not statistically significant, suggests the possibility of differences. Collectively, these findings highlight the complex interplay between genetic mutations and functional adaptations in bacteria.

### 3.12 Comparative Genomic Analysis of Resistance Genes and Virulence Factors

**Figure 9:**
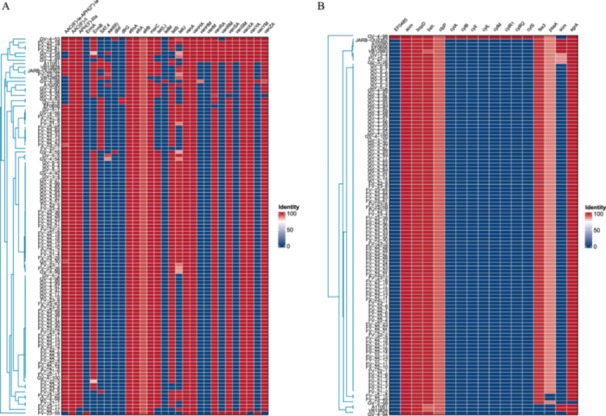
Comparative genomic analysis of resistance genes and virulence factors between the outbreak and Indian (A10290) strains. (A) Illustrates the similarity in resistance genes between the outbreak and Indian (A10290) strains, with unique traits noted in the outbreak strain. (B) Shows a high degree of overlap in virulence factors, suggesting a common pathogenic potential.

To illustrate the high degree of genetic similarity in both resistance genes and virulence factors between the outbreak and Indian A10290 strains, we also investigated the potential impact of plasmid content on strain differentiation. Figure 9A shows a comparative analysis of the resistance genes between the outbreak and Indian A10290 strains. The analysis revealed a high degree of similarity in resistance genes, including vancomycin‐resistance genes such as *vanA* and *vanHA*, indicating a shared genetic basis for antibiotic resistance. Despite the overall consistency, the outbreak strain exhibited unique characteristics within its resistance gene profile, suggesting a distinct evolutionary trajectory or selective pressure. Notably, Figure 9A reveals certain differences in the resistance genes for other antibiotics. These differences may result from the acquisition of plasmids or genetic fragments from other bacteria during the spread of the strain, leading to some recombination.

Figure 9B shows a comparative analysis of the virulence factors between the two strains. As shown in Figure 9A, the virulence factors were largely conserved among the strains, which is crucial for the pathogenic potential of the outbreak strain. Although both the resistance genes and virulence factors showed a high level of identity, the differences observed, particularly in the resistance genes of the outbreak strain, may be attributed to variations in the plasmids carried by the strains. Plasmids are important vectors for the dissemination of resistance and virulence traits among bacteria.

### 3.13 Molecular Biology Testing for Early Detection of the Outbreak Strain

**Figure 10:**
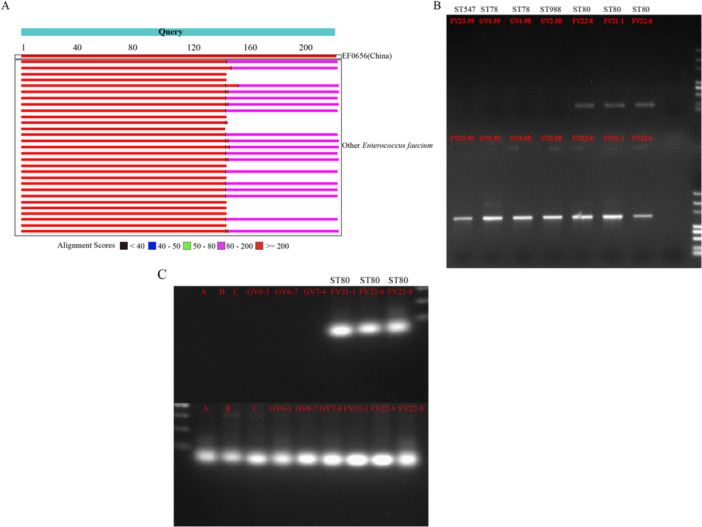
Molecular PCR testing for early screening of the outbreak strain. (A) The primer pair BLAST assembly results. (B) Specific PCR results are displayed. The samples are listed from left to right as follows: FV23‐59, GV4‐59, GV4‐98, GV2‐58, FV22‐8, FV21‐1, and FV22‐6. The samples in the lower portion were amplified with universal 16S primers (27F and 1492R), whereas the samples in the upper portion were amplified with primers specific to the ST80 strain associated with this VR‐Efm outbreak. VR‐Efm, vancomycin‐resistant *Enterococcus faecium*. (C) Extended specificity validation of the ST80‐specific PCR assay. The samples in the upper portion were amplified with the outbreak‐specific primers, and the samples in the lower portion were amplified with universal 16S rRNA primers as controls. Lanes from left to right: clinical Enterococcus isolates A, B, and C (unrelated to the outbreak); repository isolates GV6‐3, GV6‐7, and GV7‐4 (not part of the outbreak cohort); outbreak ST80 strains FV21‐1, FV22‐6, and FV22‐8.

To establish a specific detection method for the outbreak ST80 clone, we designed a PCR primer pair targeting its unique genomic region. In silico analysis confirmed the specificity of this target within the outbreak‐related strains (Figure 10A). We initially validated the primers using a panel of isolates with known sequence types (Figure 10B). The primers specifically amplified ST80 outbreak strains (FV22‐8, FV21‐1, FV22‐6) but yielded no amplification for non‐ST80 strains, including ST78 types GV4‐59 and GV4‐98, ST988 type GV2‐58, and ST547 type FV23‐59. DNA integrity for all samples was confirmed using universal 16S rRNA primers. To further evaluate the assay's specificity, we tested an extended validation panel (Figure 10C). This panel incorporated three clinical *Enterococcus* isolates (A, B, C) that were identified by MALDI‐TOF mass spectrometry as unrelated to the outbreak, along with three *Enterococcus* isolates (GV6‐3, GV6‐7, GV7‐4) from our hospital's repository that were not associated with the current outbreak. No specific amplification was observed in any of these six negative control samples, whereas the positive controls (outbreak ST80 strains) produced the expected amplicon. These data demonstrate that the PCR assay can specifically identify the epidemic ST80 clone within the context of the diverse negative controls tested.

### 3.14 Pan‐Genomic Analysis

To assess and compare the genetic composition and stability of the aforementioned strains, we conducted a pan‐genomic analysis of the VR‐Efm strains from the current outbreak and evolutionarily similar strains from India (A10290, BA12993, BA17124, BA7523, and VB13828) and Japan (JARB). The results showed that the curves corresponding to the core and private genes eventually plateaued, leading us to observe that the pan‐genome curve reached a plateau within the set of outbreak strains analyzed in this study. They are evolutionarily and functionally stable in terms of gene functionality (Figure S1A). This analysis showed that the pan‐genome curve reached a plateau for the set of closely related outbreak and reference strains analyzed here (Figure S1B,C), indicating a stable gene content within this specific cluster.

## 4 Discussion

*Enterococcus* is an opportunistic pathogen with the ability to reside in the human gastrointestinal tract for extended periods without causing any apparent infectious symptoms. Moreover, it can persist in hospital settings (Bezdicek et al. 2023). Over the past few decades, there has been a notable increase in the prevalence of acquired antimicrobial resistance in *Enterococcus* species (Kjær Hansen et al. 2021b), including VR‐Efm. Consequently, there is an imperative need for proactive surveillance to effectively prevent the rise and spread of VR‐Efm (Moosavian et al. 2018). Recent reports of enterococcal strains showing resistance to a growing array of antibiotics, including vancomycin, a critical last line of defense against gram‐positive infections, have been alarming (Ekwanzala et al. 2020). We conducted a multicenter study to investigate the prevalence of gastrointestinal VR‐Efm carriers among hospital patients in Guangzhou and Foshan by analyzing the molecular epidemiology of VR‐Efm. The clinical information and characteristics of the VR‐Efm outbreak in Guangdong were particularly notable, highlighting a considerable challenge for hospital‐acquired infections. VR‐Efm infections were detected in clinical samples, including urine, abdominal fluid, and wound swabs. The diversity of the affected patient population and sample types highlights the broad impact and complexity of curbing the spread of VR‐Efm in healthcare settings. To the best of our knowledge, this is the largest systematic study conducted in China, covering a wide range of departments, to investigate the VR‐Efm outbreak. The results are further strengthened by the multicenter design.

Glycopeptide resistance in *Enterococci* is usually mediated by *van* gene clusters, of which *vanA* and *vanB* are the most commonly reported worldwide (Rios et al. 2020). Consistent with previous studies, our research indicates that the *vanA* gene remains the predominant determinant of resistance during this outbreak of VR‐Efm. Generally, the *vanA* genotype is characterized by an acquired high level of resistance to both vancomycin and teicoplanin, whereas the *vanB* genotype is characterized by variable acquired levels of resistance to vancomycin, but not teicoplanin (Liu et al. 2022). *VanA*‐type vancomycin resistance is characterized by the transposon Tn1546‐mediated ability of bacteria to synthesize cell walls through an alternative pathway, thereby conferring high‐level resistance to vancomycin. *VanA‐*type VR‐Efm outbreaks have been reported worldwide (Cassone et al. 2023b), and costly countermeasures are required to prevent the transmission of VR‐Efm in hospitals. All isolates in our study were examined for multidrug resistance. Streptomycin demonstrated complete in vitro activity, and linezolid exhibited notable efficacy, against the VR‐Efm strains (99%).

Outbreaks of nosocomial infections caused by VR‐Efm have recently increased in Europe and Australia because *E. faecium* belongs to clonal complex 17 (CC17). *E. faecium* CC17 adapts to hospital environments by acquiring antimicrobial resistance genes, pathogenic islands, and other mobile genetic elements. Outbreaks were caused by *E. faecium* CC17 with the vancomycin‐resistance genes *vanA* and *vanB*. Both *vanA* and *vanB* encode ligases that contribute to vancomycin resistance by substituting the d‐Ala‐d‐Ala peptide in peptidoglycans with d‐Ala‐d‐Lac, which has been reported worldwide. However, the *vanA* carriers are more prevalent (Alotaibi et al. 2022). In 2019, a widespread outbreak of VR‐Efm ST1421 (CC17) occurred in Japan and extended beyond the jurisdiction of the health authorities (Ahmed et al. 2020). A target for the number of patients with VR‐Efm infection was set for the first time in Japan. However, by 2024, a large VR‐Efm outbreak was still occurring in Hiroshima. In that study, 103 VR‐Efm isolates were analyzed using WGS, with 93 identified as *E. faecium* ST80, which constituted the vast majority, consistent with the results of our study. In the present study, MLST revealed four STs among the 101 strains: 97 were identified as ST80, two as ST78, one as ST988, and one as ST547. To some extent, this suggests horizontal transfer of the van gene cluster among *E. faecium* strains. Overall, these data indicate that clonal expansion and the horizontal transfer of resistance genes contribute to the increased prevalence of VR‐Efm in hospitals.

VR‐Efm poses significant clinical challenges, particularly in immunocompromised patients. Effective infection control requires rigorous protocols, including surveillance, isolation, and stewardship. Previous studies on this topic have neglected the detection of asymptomatic carriers and lacked preserved samples for retrospective studies. The economic impact of outbreaks is substantial, affecting the healthcare infrastructure and public health. Hence, an investment in sophisticated control measures and innovative treatments is crucial to curb increasing societal costs and avert the further spread of VR‐Efm through enhanced screening efforts.

Through genomic analysis, our study sheds light on the roles of specific molecular markers in conferring antibiotic resistance to VR‐Efm, thereby offering potential targets for the development of future antimicrobial therapies. Proteins, such as 16S rRNA (cytosine(1402)‐N(4))‐methyltransferase, sensor histidine kinase, LCP family proteins, and ABC transporter ATP‐binding proteins, are crucial for physiological regulation, virulence, and the emergence of antibiotic resistance in bacteria. Genomic analysis pinpointed variations in specific molecular factors (e.g., RsmH, histidine kinase) whose broader roles in drug resistance and adaptation are documented, prompting a focused yet cautious evaluation of their potential relevance to this outbreak.

Through genomic analysis, our study identified specific SNPs within genes encoding proteins of known importance in bacterial adaptation, such as the 16S rRNA methyltransferase (RsmH), a sensor histidine kinase, an LCP family protein, and an ABC transporter ATP‐binding subunit. It is crucial to note that the functional consequences of these specific SNPs remain experimentally unvalidated in this study; they should therefore be regarded primarily as genetic markers of the outbreak lineage. However, the broader roles of these gene products in antibiotic resistance and stress response are supported by literature. For instance, 16S rRNA methyltransferases can confer resistance to ribosome‐targeting antibiotics like tetracyclines (Zou et al. 2020). Two‐component systems are key signaling modules for environmental adaptation (Ishii and Eguchi 2021). LCP proteins are involved in cell wall integrity and virulence regulation (Rajaei et al. 2022), and ABC transporters often mediate antibiotic efflux (Pan et al. 2020). Thus, while our data highlight these loci as sites of variation, the hypothesis that these specific mutations directly contributed to the outbreak strain's resistance or fitness requires future functional investigation.

This study has several limitations. First, and most critically, phylogenetic analysis alone cannot definitively establish the geographic origin or direction of transmission. While our data show a close genetic relationship between the outbreak ST80 clone and lineages from India and Japan, this pattern is consistent with multiple epidemiological scenarios and does not resolve the outbreak's source or spread pathway. Second, the isolates were primarily from two hospitals in Guangdong Province, which may not fully represent the broader epidemiological context. Third, the genomic analysis relied on short‐read sequencing data, which constrains the precise resolution of complex structural variants. Fourth, the functional link between the specific genetic variations identified (e.g., SNPs) and phenotypic outcomes (e.g., enhanced resistance) remains experimentally unvalidated. Finally, the newly developed PCR assay requires external validation with a broader and more diverse set of samples to confirm its generalizability.

The identification of a unique genomic region enabled the development of a PCR assay targeting the outbreak ST80 clone. Our validation indicates that this assay can differentiate the outbreak strain from other VR‐Efm sequence types and from unrelated clinical Enterococcus isolates in the tested collection. This suggests its potential utility for rapid molecular screening and outbreak tracing of this specific clone within the affected healthcare network. It is important to emphasize that the current validation is based on a retrospective strain set. The clinical sensitivity and specificity of this assay, particularly when applied directly to patient specimens, require evaluation in prospective studies. Furthermore, the observed specificity is tied to the genetic context of the outbreak lineage. Comprehensive testing against a broader and more diverse collection of global ST80 strains is necessary to fully define its epidemiological applicability.

## Author Contributions

Conceptualization: Y.G., N.Q., J.H., and C.Z. Methodology: Y.G., C.Z., T.Z., K.W., and Z.S. Data analysis: Y.G., C.Z., and T.Z. Writing – original draft: Y.G. Writing – review and editing: N.Q., J.H., and Y.Z. Supervision: N.Q., and C.Z. Funding acquistion: N.Q., Y.G., J.H., and C.Z.

## Ethics Statement

The authors have nothing to report.

## Conflicts of Interest

The authors declare no conflicts of interest.

## Supporting information

Supporting File 1

Supporting File 2

Supporting File 3

Supporting File 4

Supporting File 5

Supporting File 6

Supporting File 7

## Data Availability

All sequencing data have been deposited with the National Center for Biotechnology Information (NCBI) under BioProject Accession ID: PRJNA1152711 (https://www.ncbi.nlm.nih.gov/bioproject/PRJNA1152711/). All other data supporting the findings of this study are available within the article and its supplementary materials.
