# Supplementary figures and images for: Genomic Insights Into a Hospital‐Acquired High‐Risk Vancomycin‐Resistant Enterococcus faecium Outbreak in Guangdong, China
Source: Microbiologyopen. 2026 Apr 13;15(2):e70288. doi: 10.1002/mbo3.70288 (PMC13076189; doi:10.1002/mbo3.70288)

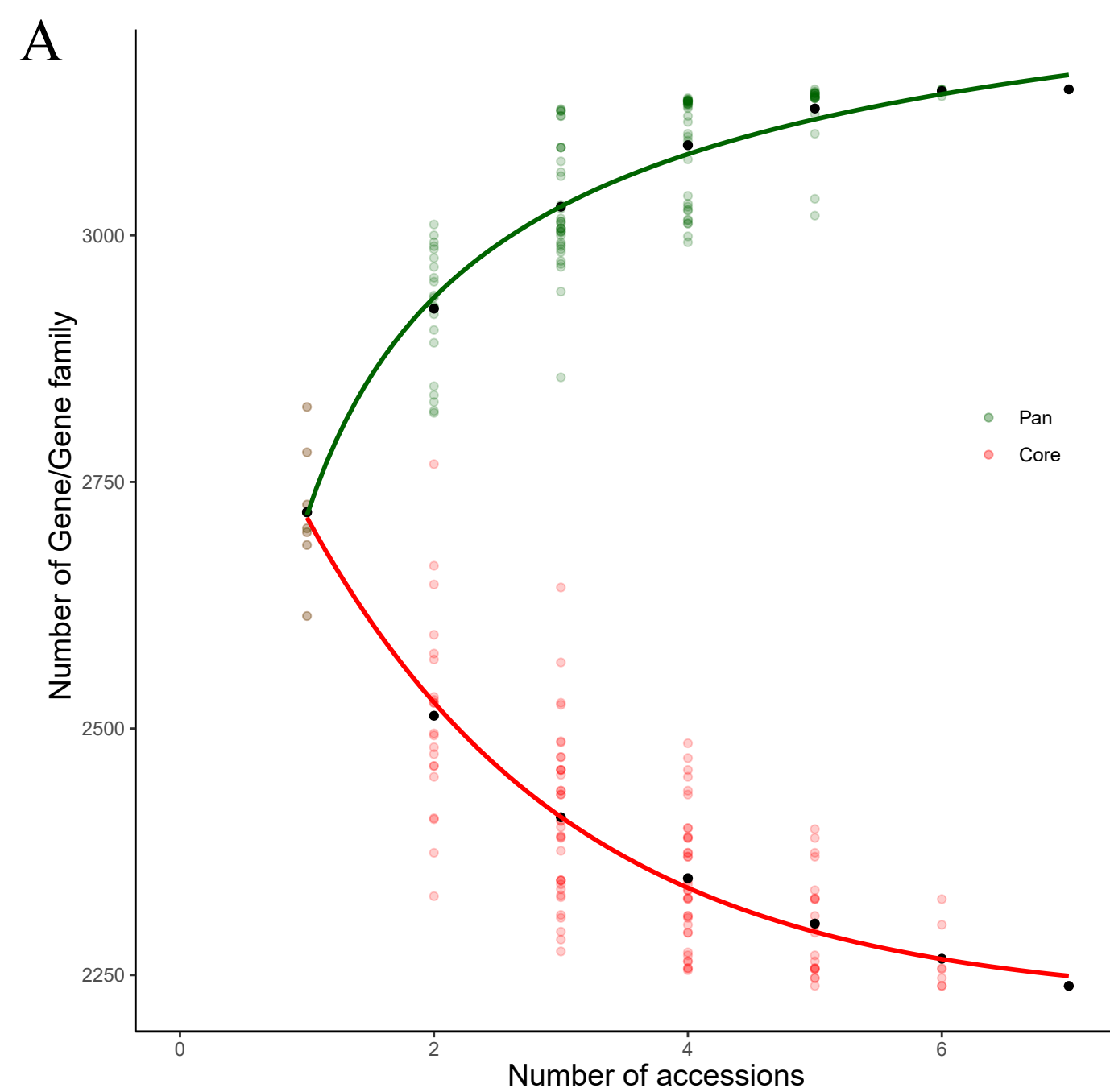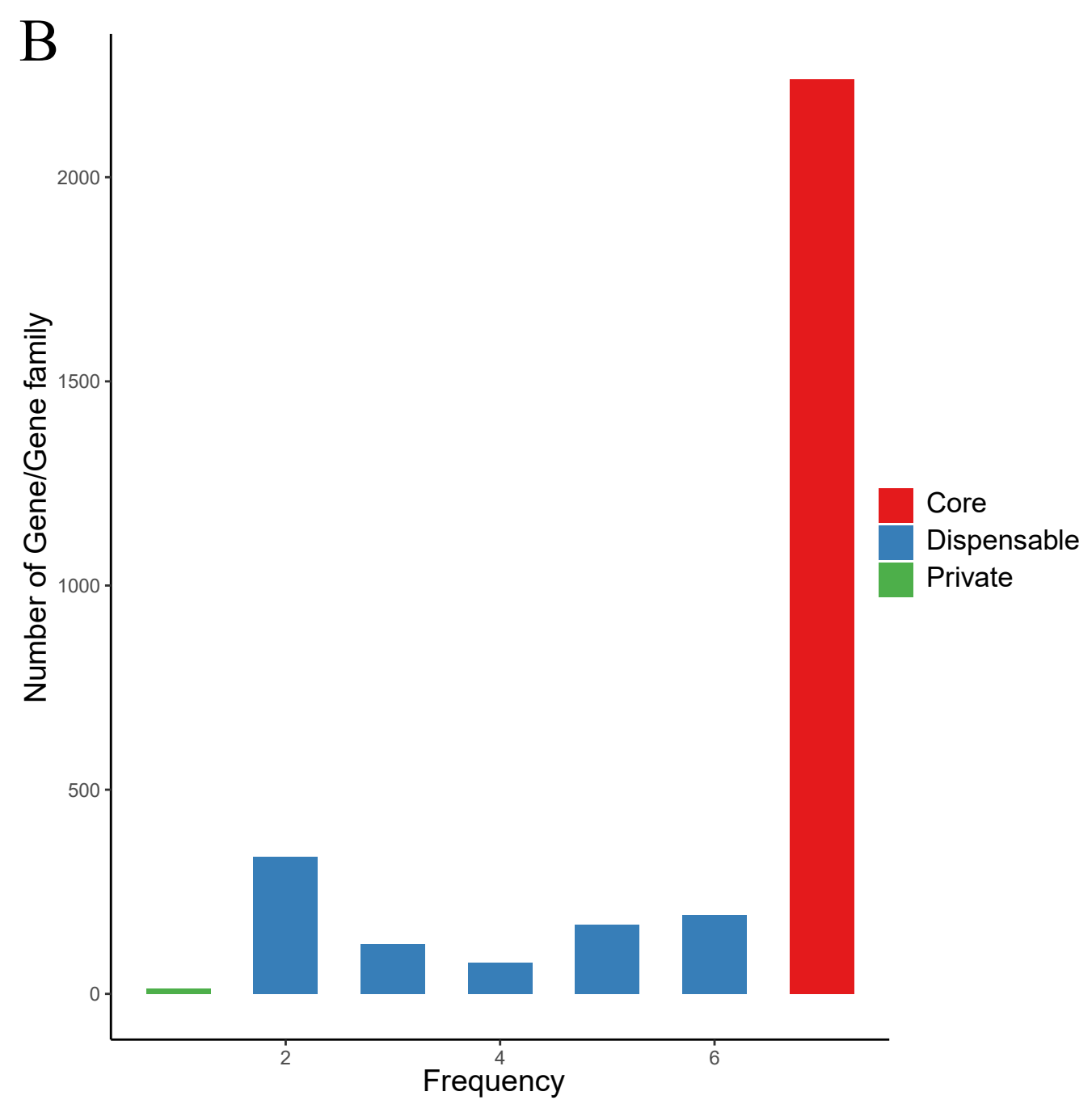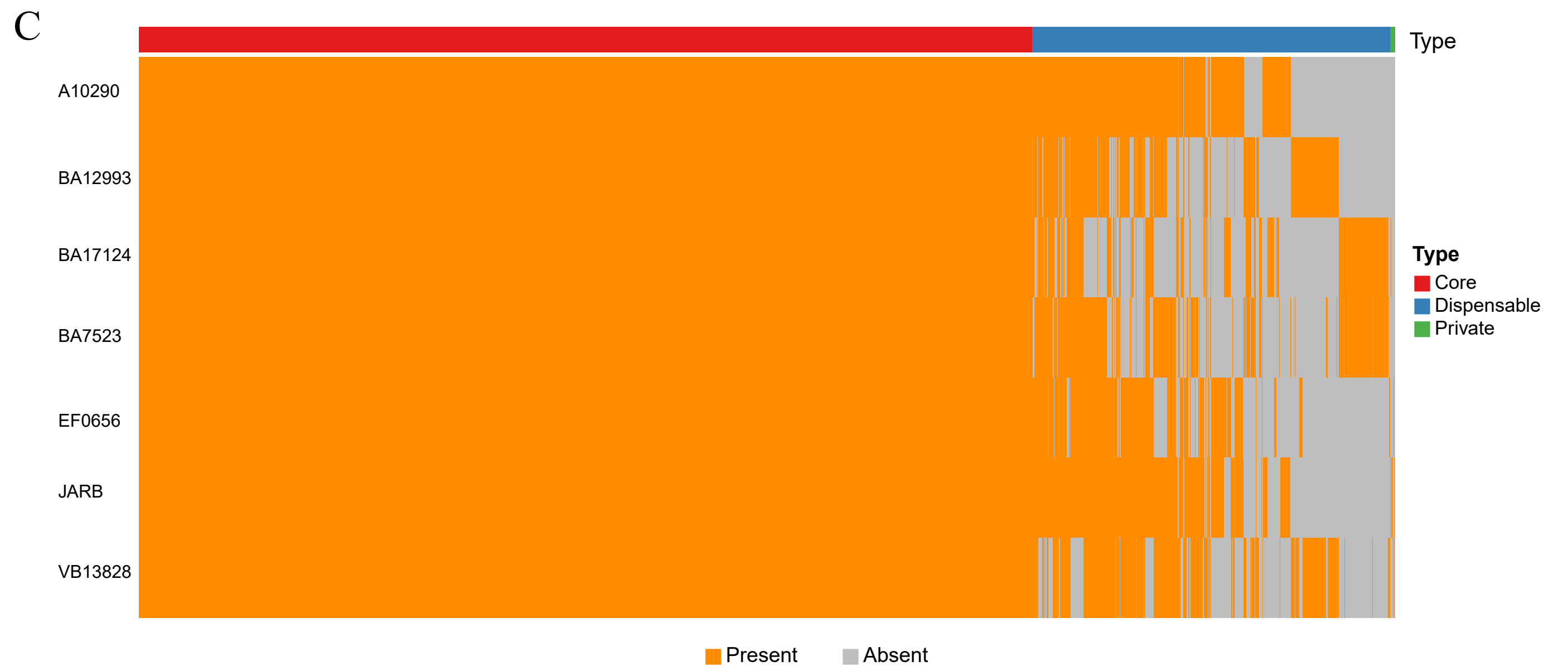

Supplement: Supplementary file 1 — Supporting File 1 [file MBO3-15-e70288-s006.pdf]

Rate=2.58, MRCA=2021.01,  $R^2=0.83$ ,  $p<0.0001$

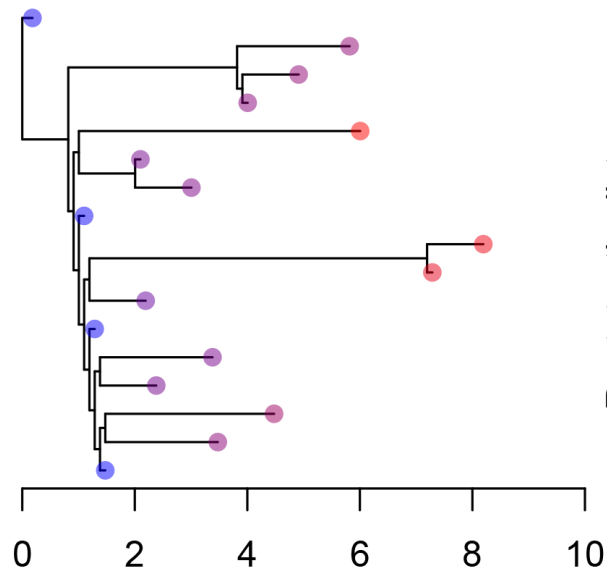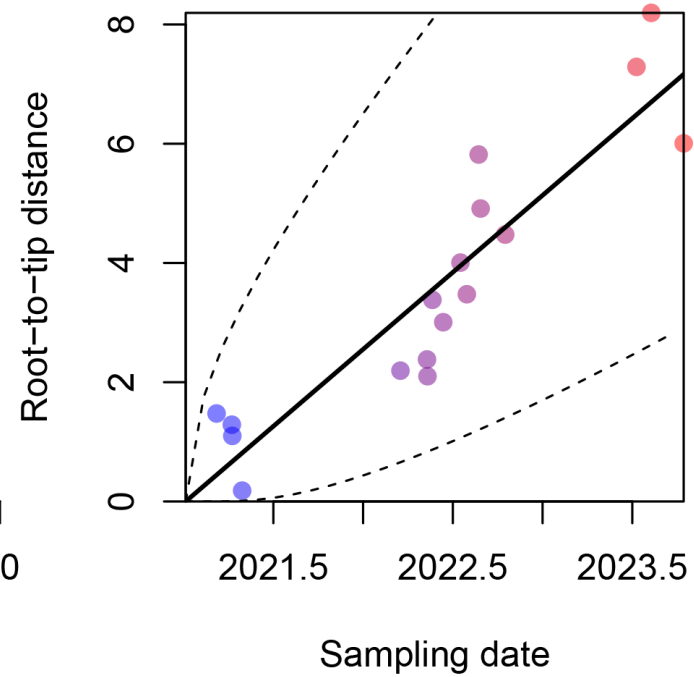

Supplement: Supplementary file 2 — Supporting File 2 [file MBO3-15-e70288-s004.pdf]
