# Supplementary material for: Genomic Insights Into a Hospital‐Acquired High‐Risk Vancomycin‐Resistant Enterococcus faecium Outbreak in Guangdong, China
Source: Microbiologyopen. 2026 Apr 13;15(2):e70288. doi: 10.1002/mbo3.70288 (PMC13076189; doi:10.1002/mbo3.70288)
Supplement: Supplementary file 3 — Supporting File 3 [file MBO3-15-e70288-s005.pdf]

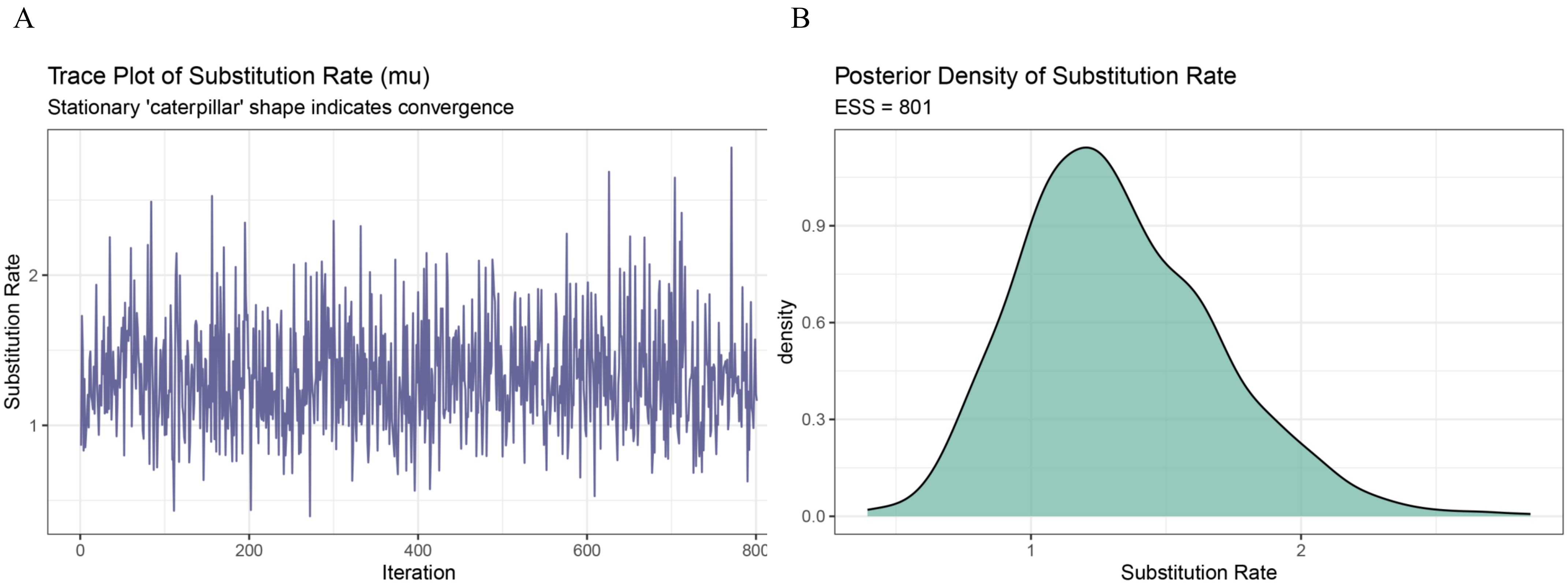
A
Trace Plot of Substitution Rate (mu)
Stationary 'caterpillar' shape indicates convergence
Substitution Rate
2
1
0
200
400
600
800
Iteration
B
Posterior Density of Substitution Rate
ESS = 801
density
0.9
0.6
0.3
0.0
1
2
Substitution Rate
