# Supplementary material for: Genomic Insights Into a Hospital‐Acquired High‐Risk Vancomycin‐Resistant Enterococcus faecium Outbreak in Guangdong, China
Source: Microbiologyopen. 2026 Apr 13;15(2):e70288. doi: 10.1002/mbo3.70288 (PMC13076189; doi:10.1002/mbo3.70288)
Supplement: Supplementary file 7 — Supporting File 7 [file MBO3-15-e70288-s001.pdf]

广州医科大学附属第一医院科研项目审查伦理委员会
临床研究会议审批件

受理号：ES-2023-057-01

<table>
<tr><td>项目名称</td><td colspan="5">《中国感染病原菌规范化分层监测体系建立与药物敏感性和耐药性现状调查——华南地区感染病原敏感性与耐药性监测调研》</td></tr>
<tr><td>申办者</td><td colspan="2">中国医学科学院北京协和医院</td><td>项目负责人</td><td colspan="2">卓超</td></tr>
<tr><td>审查日期</td><td colspan="2">2023-04-27</td><td>审查地点</td><td colspan="2">广州医科大学附属第一医院沿江院区新住院大楼 30 楼 3008 会议室</td></tr>
<tr><td>审查类别</td><td colspan="5">初始审查</td></tr>
<tr><td rowspan="3">表决情况</td><td colspan="5">委员人数 15 人；出席人数 9；回避人数 0；弃权人数 0；</td></tr>
<tr><td>同意</td><td>作必要的修正后同意</td><td>作必要的修正后重审</td><td>不同意</td><td>终止或暂停已批准的试验</td></tr>
<tr><td>9</td><td>0</td><td>0</td><td>0</td><td>0</td></tr>
<tr><td>审查结论</td><td colspan="5">同意</td></tr>
<tr><td colspan="6">审查意见：
同意按照提交文件开展项目/研究。</td></tr>
<tr><td colspan="6">伦理委员会主任委员签字：
日期：2023-05-05
广州医科大学附属第一医院科研项目审查伦理委员会（盖章）</td></tr>
<tr><td>备注</td><td colspan="5">1.修改后同意/重审项目，应将修改后文件及时反馈伦理委员会，进行进一步审查。
2.不同意/终止或暂停项目，批件发出 2 周内可向伦理委员会就有关事项做出解释或提出申诉。
3.临床试验应严格按照本伦理委员会批准的文件执行。在试验实施过程中，如对试验方案、知情同意书等文件做任何修改，应及时向本伦理委员会提交变更申请，补充更新文件，经伦理委员会重新审查批准后，方可执行。
4.发生严重不良事件及可能影响风险受益的任何事件和新信息须及时报告本伦理委员会。
5.定期/年度跟踪审查项目，于到期后 1 周内提交试验进度情况报告。如有不依从/违背方案或暂停/提前终止的试验项目，应及时以书面文件告知本伦理委员会。试验结束后，须及时向伦理委员会提交结题报告。
6.本批件有效期为 12 个月（自批准之日起）。若在有效期内未启动项目，则本批件自动终止。
7.本委员会依据 GCP 和国家法规以及 ICH-GCP 的要求操作。
8.凡涉及中国人类遗传资源管理办公室批准的研究项目，需获得遗传办公室批准后才能开始研究。</td></tr>
<tr><td colspan="6">联系方式：广州市桥中中路 28 号</td></tr>
<tr><td colspan="4">电话：020-81566265　　传真：020-83389471</td><td colspan="2">联系人：张晓露</td></tr>
</table>

审查文件清单：

1.科研伦理审查申请表

2.研究方案--华南地区感染病原敏感性与耐药性监测调研(版本日期:2023-03-22 版本号:V1.0)

3.符合豁免知情同意说明(版本日期:2023-03-22 版本号:V1.0)

4.主要研究者专业履历

5.《华南地区感染病原敏感性与耐药性监测调研》项目任务书

6.《中国感染病原菌规范化分层监测体系建立与药物敏感性和耐药性现状调查》项目任务书

7.牵头单位中国医学科学院北京协和医院伦理批件

8.多中心清单、单位简介和主要研究者简历
